# Degradation of PVC waste into a flexible polymer by chemical modification using DINP moieties†

**DOI:** 10.1039/c9ra05081g

**Published:** 2019-09-13

**Authors:** Lihui Lu, Shogo Kumagai, Tomohito Kameda, Ligang Luo, Toshiaki Yoshioka

**Affiliations:** Graduate School of Environmental Studies, Tohoku University 6-6-07 Aoba, Aramaki-aza, Aoba-ku Sendai Miyagi 980-8579 Japan yoshioka@env.che.tohoku.ac.jp +81-22-795-7212 +81-22-795-7212; College of Life Science, Shanghai Normal University 100 Guilin Road Shanghai 200234 China luo_ligang@yahoo.com

## Abstract

In consideration of the toxicity and high migration capacity of plasticizers, the possibility to obtain flexible PVC *via* chemical modification of PVC was investigated for feedstock recycling. In this work, some Cl atoms of PVC were substituted with fragments of the common plasticizer DINP (diisononyl phthalate) in the presence of K_2_CO_3_ (potassium carbonate) or DIEA (*N*,*N*-diisopropylethylamine), and the simultaneous elimination of PVC was suppressed. ^1^H NMR (^1^H nuclear magnetic resonance spectroscopy) and ^1^H–^1^H COSY (^1^H–^1^H correlation spectroscopy) were used to evaluate the substitution while a novel method of calculating the substitution and elimination ratios was developed using a combination of ^1^H NMR and elemental analysis. A maximum substitution rate of 35.7% was achieved using thiophenol as a nucleophile in the presence of DIEA, while the corresponding elimination of HCl was just 4.4%. In addition, the thermal stability of the modified PVCs was very close to that of pure PVC, which suggested that the main characteristics of PVC were preserved. Moreover, the *T*_g_ values of all the modified PVCs were less than that of PVC, which means it is feasible to improve the plasticity of PVC *via* substituting some Cl on PVC with DINP moieties. Therefore, an alternative approach for feedstock recycling of PVC by chemical modification was developed in this work.

## Abstract 2

We propose a chemical modification method to produce flexible PVC with DINP moieties.

## Graphical abstract

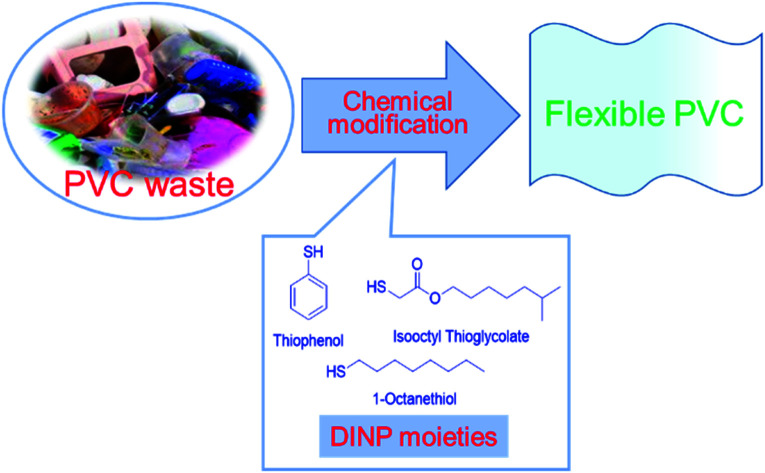

## Introduction

Plastics are inexpensive, lightweight, durable and corrosion-resistant, with high thermal and electrical insulation properties. The diversity of polymers and the versatility of their properties are used to make a vast array of products that bring medical and technological advances, energy savings and numerous other societal benefits.^1–3^ A world without plastics seems unimaginable today. However, plastic waste is a growing social problem, because of loss of natural resources, depletion of landfill space, and environmental pollution, especially for the marine environment.^4,5^ Therefore, fuel and feedstock recycling of plastic wastes has attracted widespread attention around the world.

As one of the most important chlorinated plastics, PVC has been widely used in manufacturing pipes,^3^ cable insulation,^4^ films,^5^ building materials,^6^ medical materials^7^ and so on. However, it is a great challenge to treat with PVC waste due to its chlorine content of *ca.* 58%. Nowadays, the treatment of PVC was suffered on the products of corrosive HCl and harmful chlorinated organic during pyrolysis treatment, which was limited various recycling demands.^8^ Therefore, a series of strategy were investigated on the migration of Cl, such as thermal degradation,^4,9–11^ chemical modification,^12–14^ hydrothermal process in supercritical or subcritical water,^15–18^ near-critical methanol process for dechlorination of PVC and additives recovery,^19^ and near-critical aqueous ammonia process.^20^ Among them, chemical modification of PVC is a famous technique that allows addition of new functionalities to PVC while maintaining its primary characteristics by substituting some Cl atoms with various nucleophilic reagents.^21–24^ In previous study, the properties of gas transport,^21^ luminescence,^22^ antibacterial,^23^ and plasticity^24^ have been improved by grafting different functional groups to substitute Cl atoms on PVC. In particular, P. Jia *et al.* investigated self-plasticization of PVC materials *via* chemical modification with phosphorus containing castor oil based derivatives,^14^ cardanol groups,^24^ aminated tung oil methyl ester,^25^ and mannich base such as cardanol butyl ether^13^ and waste cooking oil methyl ester.^26^ These findings transformed the traditional plastic processing technology to obtain cleaner production of internally plasticized PVCs. Therefore, this technique has a great potential to be used for PVC recycling because it can convert waste PVC into value-added materials with improved properties, which can be referred to “upgrade recycling”.

Without plasticizer, PVC is a rigid polymer at room temperature. Nevertheless, PVC products are normally obtained from the combination of PVC with additives which provides our desired properties to the material.^27^ The plasticizers, which act as spacer between molecules of the polymer, are mainly used to reduce the melt viscosity, to decrease the transition temperature or to lower the elastic modulus of PVC as it weakens the intermolecular bonds of the polymer which facilitate its processing.^27^ The most commonly used plasticizer were terephthalate species, such as diisononyl phthalate (DINP), diethylhexyl phthalate (DEHP), diisobutyl phthalate (DIBP), di-*n*-butyl phthalate (DBP), and diisodecyl phthalate (DIDP).^28^

**Fig. 1:**
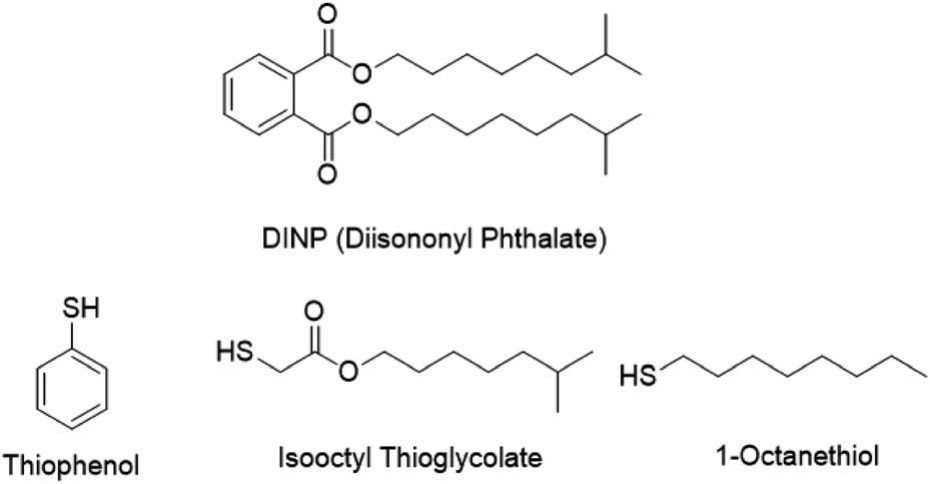
Structures of DINP and the nucleophiles investigated in this work.

However, most of these plasticizers have been banned in the last decades due to their high migration capacity and strong toxicity.^29,30^ Therefore, the possibility to produce flexible PVC by chemical modification using common plasticizer DINP moieties as nucleophiles (Fig. 1), which was investigated in this work for feedstock recycling. The obtained products are environmentally-friendly while compared with a mixture of PVC and plasticizer. However, the main problem is the elimination reaction simultaneously occurring with a strong base such like sodium hydroxide (NaOH),^31^ which have effect on the properties of PVC and causes a blackish color, which will narrow the applicability of the recycled PVC. As a result, the effect of potassium carbonate (K_2_CO_3_) and *N*,*N*-diisopropylethylamine (DIEA) were investigated as a weak inorganic base and organic base on substitution and elimination, respectively. The substitution was evaluated by ^1^H nuclear magnetic resonance (^1^H NMR). Moreover, a novel method of calculating the substitution and elimination rates based on ^1^H NMR and elemental analysis was developed, which is simpler and more accurate than the methods used in previous study.^32^

## Results and discussion

### Confirmation of the substitution of PVC with thiophenol

At first, the FT-IR spectra were employed to investigate the structure between PVC and the modified PVC with thiophenol (Fig. S1†). The sharp peaks of PVC at 2969, 2920, 1436 and 606 cm^−1^ are belong to the alkane C–H stretching vibration, –CH_2_ deformation vibration and C–Cl stretching vibration, respectively.^13,33^ Compared to the curve of PVC, it was observed that several new peaks at 3075, 1575 and 1552 cm^−1^ were appeared, which are attributed to C–H stretching vibration of arene and the skeleton vibration of benzene ring, respectively.^34^ This was suggested that the thiophenol group was successfully grafted on PVC backbone, which means the nucleophilic substitution of PVC and thiophenol was efficient in the presence of K_2_CO_3_ as a base.

**Fig. 2:**
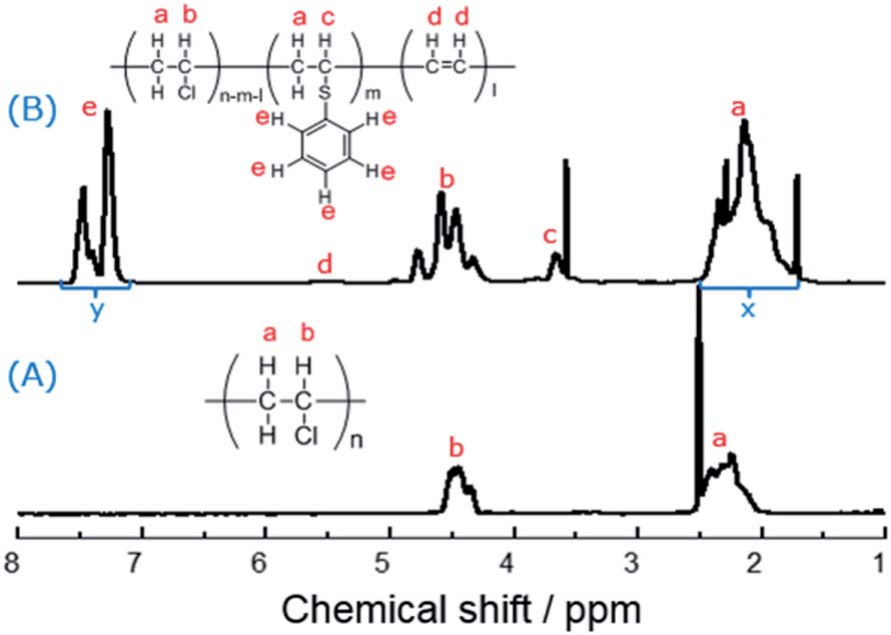
^1^H NMR spectra of PVC and the modified PVC using thiophenol as a nucleophile (400 MHz, THF-d_8_, 329.9 K. Reaction conditions: 500 mg PVC, 1.0 eq. thiophenol, 0.1 eq. K_2_CO_3_, 40 °C, 50 mL DMF as solvent, 3 h).

Subsequently, the ^1^H NMR spectra was used to identify the PVC and the modified PVC with thiophenol. In Fig. 2, one of common signal of the chemical shift between 2.25–2.52 ppm is attributed to hydrogen atoms a, and the other common signal of the chemical shift between 4.35–4.52 ppm is attributed to hydrogen atom b.^22^ Compared to the spectrum of PVC (Fig. 2(A)), there were new peaks of hydrogen atoms c and e observed in Fig. 2(B). It is obviously noted that a single sharp peak of the chemical shift appeared around 3.66 ppm, which is attributed to the hydrogen atom c of the new produced group (–CH–S–) of the modified PVC with thiophenol. It was suggested that a portion of chlorine atoms were already substituted by thiophenol and the nucleophilic substitution occurred. Moreover, the signals of hydrogen atoms e are also observed at the chemical shifts between 7.25–7.50 ppm.

On the other hand, the peak of hydrogen atoms d (CH=CH) is not obviously observed in Fig. 2(B), which was suggested that the simultaneous elimination of PVC was suppressed under the reaction condition. Therefore, the rate of dechlorination was consistent with the ratios calculated from elemental analysis, which was according to the previously reported methods,^32^ and then the corresponding elimination rate could be obtained. Nevertheless, substitution ratio was calculated from their ^1^H NMR spectra. In Fig. 2(B), it was showed that the chemical shifts between 7.25–7.50 ppm is attributed to the hydrogen atoms e (five hydrogen atoms) which are from thiophenol before the reaction, while the chemical shifts between 1.95–2.35 ppm is attributed to the hydrogen atoms a (two hydrogen atoms) which are from PVC before the reaction. Therefore, the substitution ratio was defined as eqn (1):

1 Substitution ratio [%] = (*x*/*m*)/(*y*/*n*) × 100%

where *x* and *y* are the integrals of the corresponding hydrogen atoms shown in Fig. 2(B), and *m* = 5 and *n* = 2 when PVC was substituted with thiophenol. The elimination ratio was determined by subtracting the substitution ratio from the dechlorination ratio, as shown in eqn (2):

2 Elimination ratio [%] = dechlorination ratio [%] − substitution ratio [%]

**Fig. 3:**
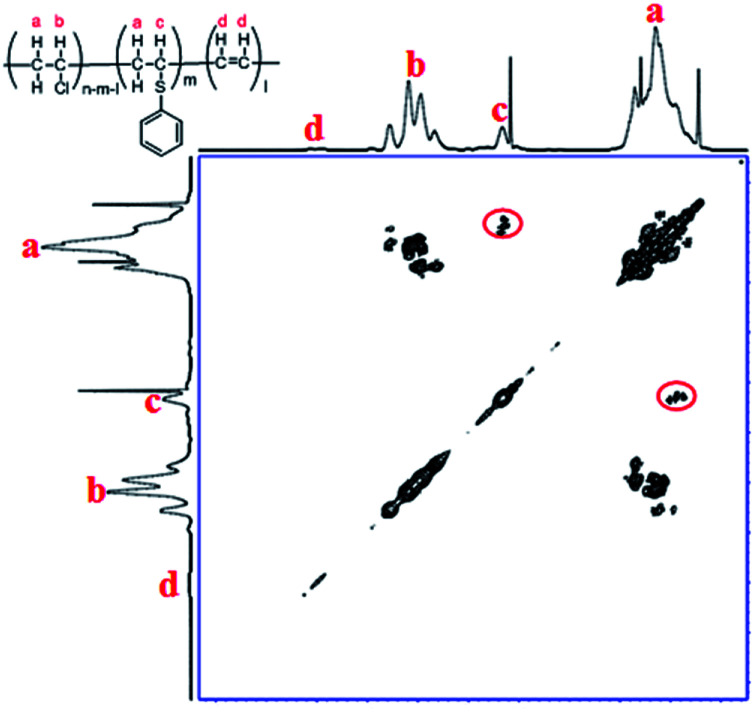
^1^H–^1^H COSY of PVC and the modified PVC with thiophenol (400 MHz, THF-d_8_, 353.0 K. Reaction conditions: 500 mg PVC, 1.0 eq. thiophenol, 0.1 eq. K_2_CO_3_, 40 °C, 50 mL DMF as solvent, 3 h).

**Scheme 1:**
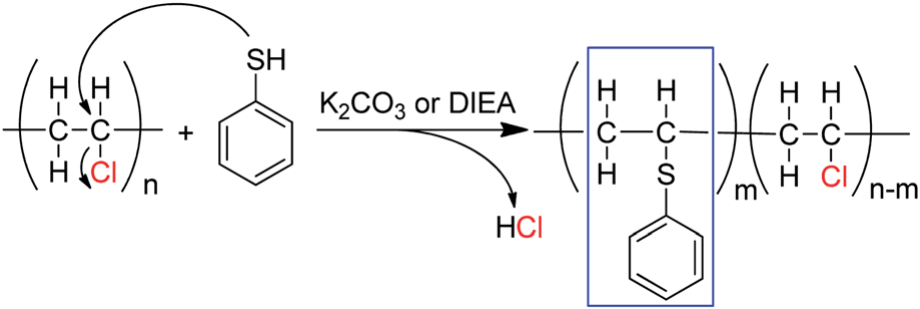
Substitution reaction of PVC with thiophenol in the presence of K_2_CO_3_ or DIEA.

To further confirm the substitution of PVC with thiophenol, ^1^H–^1^H COSY was also performed for characterization of the products. In Fig. 3, the correlation peaks (marked with red circles) between hydrogen atoms a and c are observed obviously. It was indicated that hydrogen atoms a and c were correlated each other and hydrogen atom c was bonding with the adjacent carbon of hydrogen atoms a, which was demonstrated that the structure of C_6_H_5_–S–CH–CH_2_– was successfully formed. According to the above results, it was considered that thiophenol was attacked to the α-position carbon of PVC to replace Cl atom and produce HCl, which is corresponding with the previous literature.^9,12^ Nevertheless, the produced HCl could be neutralized by the base of K_2_CO_3_ or DIEA. Therefore, one part of Cl atoms in PVC were successfully substituted by thiophenol, which is in accordance with the experimental result from ^1^H NMR spectrum in Fig. 2(B) and Scheme 1.

### Characterization of the modified PVCs

Subsequently, isooctyl thioglycolate and 1-octanethiol were conducted as nucleophilic reagents to modify the PVC sample. And the dechlorination degrees of PVC using the three different nucleophiles in DMF using K_2_CO_3_ or DIEA as bases were summarized in Table 1. When taking thiophenol as a nucleophile, the rate of dechlorination was 20.2% in the presence of K_2_CO_3_ (Table 1, entry 1), while the dechlorination rate was 40.1% with DIEA as a base (Table 1, entry 2). This was attributed to the corresponding high substitution ratios of 17.5% and 35.7%, respectively. However, when using isooctyl thioglycolate and 1-octanethiol in the presence of K_2_CO_3_, the dechlorination ratios were 7.0% (Table 1, entry 3) and 10.7% (Table 1, entry 5), respectively. This was because their corresponding substitution rates were just 6.5% and 6.3%, respectively. While using DIEA as a base, the degree of substitution for isooctyl thioglycolate was just 3.7% (Table 1, entry 4), and there was no reaction using 1-octanethiol (Table 1, entry 6), which was showed lower efficiency. Therefore, the efficiency condition of substitution was using thiophenol as the nucleophile because the nucleophilic group C_6_H_5_S– is more easily formed under the reaction condition due to the conjugation of the benzene ring. In addition, it was suggested that the effect of isooctyl thioglycolate was shown better than that of 1-octanethiol as the nucleophile when both organic and inorganic bases were used, probably due to the positive influence of electron-withdrawing carbonyl group (C=O). Furthermore, when thiophenol was the nucleophile, a better performance was obtained using the organic base DIEA, whereas the inorganic base K_2_CO_3_ was a better base when isooctyl thioglycolate and 1-octanethiol were used, as shown in Table 1. Finally, all the elimination ratios of the modified PVC were lower than their corresponding substitution ratios, probably due to the relatively weak basicity of K_2_CO_3_ and DIEA in comparison with NaOH.^17^

**Table 1:** Degrees of dechlorination, substitution and elimination for the chemical modification of PVC using different nucleophiles and bases^a^

Entry	Nucleophile	Base	Dechlorination^b^ (%, EA)	Substitution^c^ (%, ^1^H NMR)	Elimination (%)
1	Thiophenol	K_2_CO_3_^d^	20.2	17.5	2.7
2		DIEA^e^	40.1	35.7	4.4
3	Isooctyl thioglycolate	K_2_CO_3_^d^	7.0	6.5	0.5
4		DIEA^e^	5.1	3.7	1.4
5	1-Octanethiol	K_2_CO_3_^d^	10.7	6.3	4.4
6		DIEA^e^	1.0	0	1.0

a Reaction conditions: 500 mg PVC, 1.0 eq. appropriate nucleophiles, 0.1 eq. K_2_CO_3_ or 1.0 eq. DIEA, 40 °C, 50 mL DMF as solvent, 3 h.

b Results were from elemental analysis.

c Results were from ^1^H NMR analysis.

d Reaction temperature: 40 °C.

e Reaction temperature: 110 °C.

### Thermal stability of PVC and the modified PVCs

**Fig. 4:**
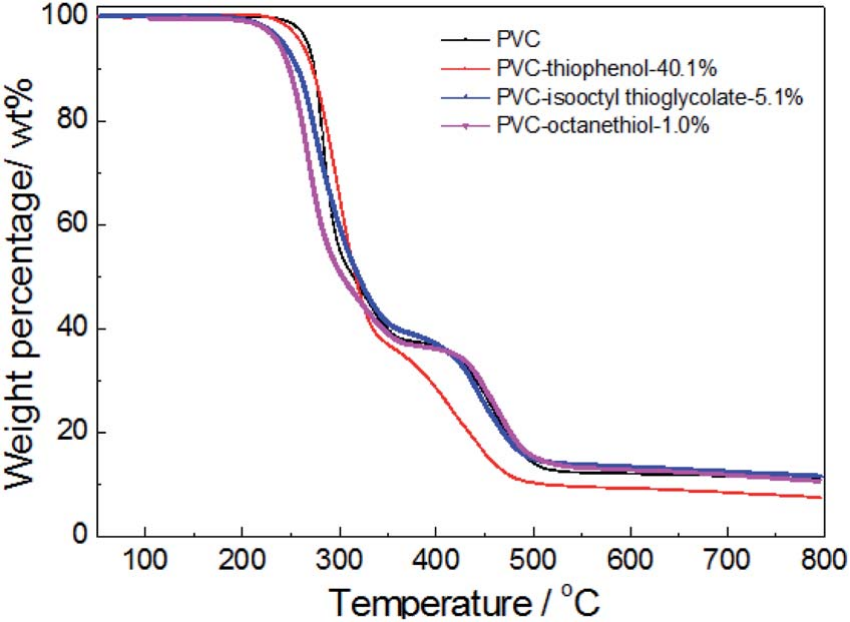
TG analysis of PVC and the modified PVCs with different nucleophiles using DIEA as a base (testing conditions: sample, 10 mg; heating temperature, 50–800 °C; heating rate, 10 °C min^−1^; 50 mL min^−1^ N_2_ flow).

For confirmation of the thermal stabilities of pure PVC and the modified PVC with different nucleophilic reagents using DIEA as a base, the thermo-gravimetric and differential thermal analysis (TG-DTA) of PVC sample were conducted in Fig. 4. At first, it was observed that the PVC used in these experiments showed a weight loss of 5 wt% at an onset temperature (*T*_D-onset_) of 269 °C. This may be led by dehydrochlorination of PVC in the temperature range of about 250–350 °C in the main reaction of the first stage, which is corresponding with the literature.^4^ However, a weight loss of 63.1 wt% was observed for unmodified PVC after the first degradation stage, suggesting that the formed polyene chain could also decomposed during the first stage. This is because that *ca.* 57 wt% of weight loss was expected from the complete elimination of PVC. As shown in Fig. 4, it was indicated that the thermal degradation of the modified PVCs with different nucleophilic groups and dechlorination ratios were showed the similar trend as that of pure PVC, which also include two distinct stages. Moreover, the product modified by thiophenol with 40.1% dechlorination ratio showed a similar degradation character to unmodified PVC during the first stage. And its *T*_D-onset_ is 261 °C and the weight loss of the first stage is 63.1%. However, it showed a different degradation behavior during the second degradation stage, the temperature range of PVC modified by thiophenol with 40.1% was around 350–480 °C, while the temperature range of the second stage on PVC was approximate 350–525 °C, which was corresponding with the cracking and decomposition of the dehydrochlorinated PVC.^4^ The possible cause might be the high substitution ratio (35.7%) and the maximum rate of dechlorination (40.1%) resulted from it. Simultaneously, the thermal stability of the products modified by isooctyl thioglycolate and 1-octanethiol with the corresponding dechlorination ratio of 5.1% and 1.0% were slightly reduced, resulting in the *T*_D-onset_ of 241 °C and 236 °C, respectively. It is worth noting that their degradation behaviors remained identical with that of pure PVC during the second stage, probably due to their low dechlorination concentration.

**Fig. 5:**
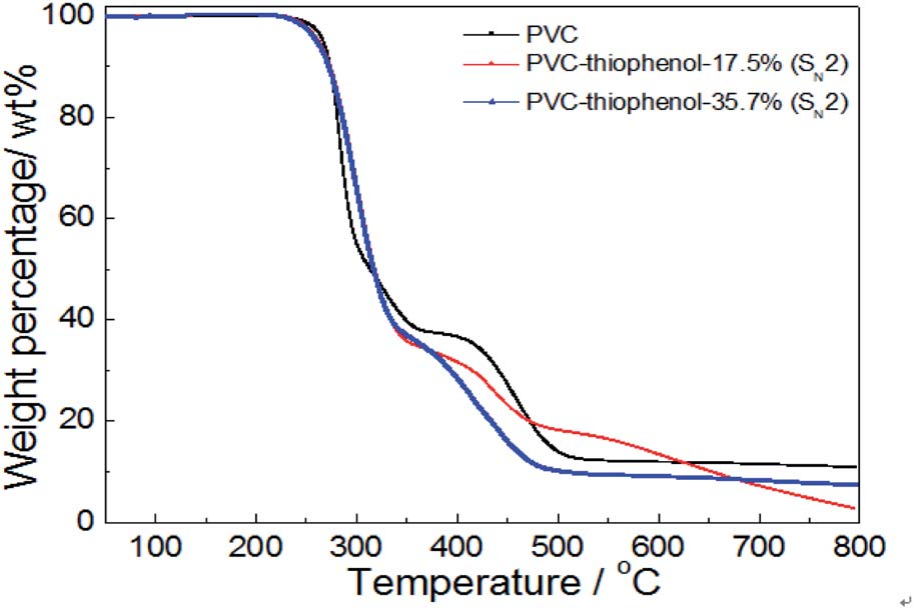
TG analysis of PVC and the modified PVCs with thiophenol using K_2_CO_3_ or DIEA as a base (testing conditions: sample, 10 mg; heating temperature, 50–800 °C; heating rate, 10 °C min^−1^; 50 mL min^−1^ N_2_ flow).

To further investigate the effect of the substituted concentration on thermal stability, the PVC substituted with thiophenol and 1-octanethiol were also conducted by TGA analysis. As shown in Fig. 5, it showed that the same degradation character was observed during the first stage (250–350 °C) for both thiophenol-substituted PVCs, even with 17.5% and 35.7% substitution rates, respectively. Nevertheless, the trends for those degradation had a significantly difference during the second stage (350–480 °C), which the sample with 17.5% substitution has loss over 20 wt% weight. It was because that the sample with 17.5% substitution ratio did not have a PVC-like character, which might be attributed to the effect of the different substituted rates or aromatic nucleophilic group (C_6_H_5_S–) itself. Then, the sample with 17.5% substitution also has a significant degradation in the third stage.

**Fig. 6:**
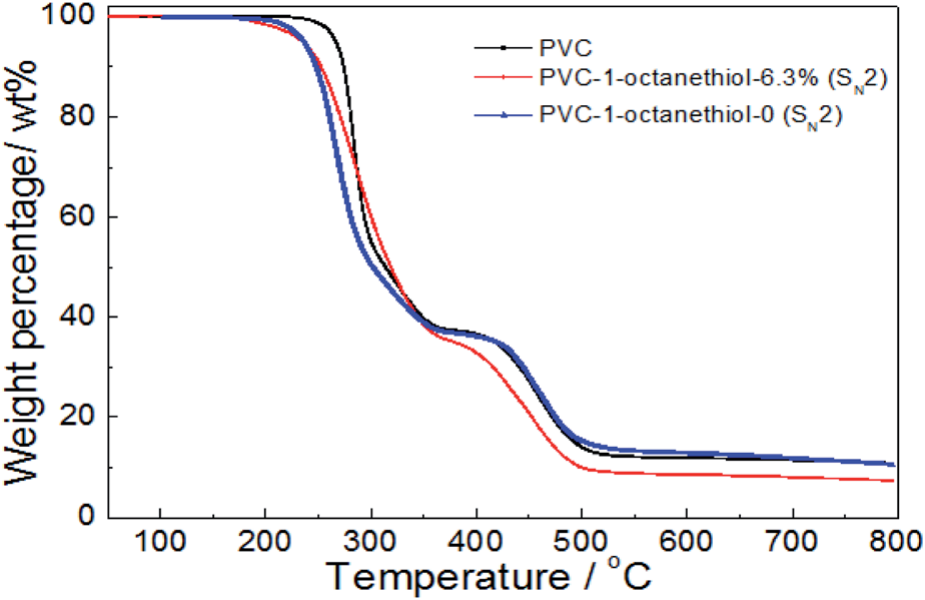
TG analysis of PVC and the modified PVCs with 1-octanethiol using K_2_CO_3_ or DIEA as a base (testing conditions: sample, 10 mg; heating temperature, 50–800 °C; heating rate, 10 °C min^−1^; 50 mL min^−1^ N_2_ flow).

In Fig. 6, the thermal stability of both 1-octanethiol-substituted PVCs slightly reduced in comparison with that of PVC during the first stage, resulting in their *T*_D-onset_ of 236 °C and 235 °C. However, the product with no substitution rate showed the similar trend as PVC during the second stage, while the modified PVC product with 6.3% substitution rate was showed easily degradable. It was implied that the thermal stability was slightly decreased on PVC modified with grafting an aliphatic nucleophilic group.

### Glass transition temperature (*T*_g_) of PVC and the modified PVCs

In addition, the glass transition temperature (*T*_g_) of PVC and modified PVCs were also detected using DSC measurements, and the results were summarized in Table 2. It was observed that the *T*_g_ of pure PVC with a stiff backbone is at around 82 °C (Table 2, entry 1). The addition of plasticizer into PVC polymer will increase the distance of PVC chains and make the macromolecular structure polymer easy to move, increase the free volume of PVC and reduce the *T*_g_.^13^ Obviously, the *T*_g_ of all the modified PVCs are less than that of pure PVC, as shown in Table 2. This indicates that internally plasticized PVCs can be obtained *via* chemical modification of PVC with appropriate nucleophilic reagents (DINP moieties). In addition, the *T*_g_ of the modified PVCs with thiophenol slightly decreased compared to pure PVC. Nevertheless, the modified PVC with 35.7% substitution ratio has a lower *T*_g_ (Table 2, entry 3) in comparison with the one with 17.5% substitution ratio (Table 2, entry 2), which suggests high substitution efficiency can be conducive to improving plasticity of PVC. Moreover, the *T*_g_ of the modified PVCs with long carbon chain nucleophiles (isooctyl thioglycolate and 1-octanethiol) distinctly decrease to 65.8 and 65.6 °C (Table 2, entries 4 and 6), respectively. It means that the effect of long carbon chain group on improving the plasticity of PVC are evidently better than that of thiophenol group. Therefore, these results illustrate that the nucleophiles of DINP moieties can play an internally plasticized effect on PVC through replacing partial Cl atoms on PVC. Besides, the factor for decreasing *T*_g_ is mainly caused that the substituting of Cl atoms with nucleophiles will increase distance between PVC chains and reduce intermolecular force.^24–26^

**Table 2:** DSC and TGA data of PVC and modified PVCs

Entry	Samples (S_N_2__)	*T*_g_ a (°C)	*T*_D_ b (°C)
1	PVC	82.1	269
2	PVC-thiophenol-17.5%	77.7	263
3	PVC-thiophenol-35.7%	75.2	261
4	PVC-isooctyl thioglycolate-6.5%	65.8	265
5	PVC-isooctyl thioglycolate-3.7%	75.6	241
6	PVC-1-octanethiol-6.3%	65.6	236

a DSC testing conditions: each sample, 10 mg; heating temperature, −50 to +120 °C; heating rate, 10 °C min^−1^; under N_2_ atmosphere.

b TGA testing conditions: sample, 10 mg; heating temperature, 50–800 °C; heating rate, 10 °C min^−1^; 50 mL min^−1^ N_2_ flow. *T*_D_: temperature of weight loss of 5 wt% for a sample.

## Experimental

### Materials

PVC powder (*M*_w_ = 68 750), K_2_CO_3_, DIEA, thiophenol, isooctyl thioglycolate and dimethylformamide (DMF) were purchased from the Wako Pure Chemical Industries, Ltd (Japan). Tetrahydrofuran (THF), 1-octanethiol and methanol were acquired from the Kanto Chemical Co., Inc. (Japan). Thiophenol, isooctyl thioglycolate, and 1-octanethiol were used as nucleophilic reagents. Water was used the ultrapure water in the experiments. All other reagents and solvents were used without further purification.

### Experimental procedure

In a type experiment, PVC powder (500 mg, 8 mmol equivalent monomer), an appropriate nucleophile (1.0 eq.), and a base (either K_2_CO_3_ (0.1 eq.) or DIEA (1.0 eq.)) were added to 50 mL of DMF in a three-neck flask while stirring. The reaction mixture was heated respectively up to 40 °C or 110 °C using a silicone oil bath for 3 h. After reaching the required reaction time, the solution was filtered and washed with methanol to obtain a precipitate. The precipitate was filtered off and dissolved in THF to remove impurities. This was poured into methanol for recrystallization, after which the white solid was filtered off and dried under reduced pressure to further analyze. Each experiment was repeated at least 2 times (in duplicate) unless otherwise noted.

### Product analysis

The purified products were analyzed by ^1^H NMR (Bruker DPX400, 400 MHz), and deuterated solvents were THF-d_8_ and DMF-d_7_. In addition, elemental analysis of the products was performed using a CHN-coder MT6 (Japan Yanaco New Science Inc.). Fourier transform infrared spectrometry (FT-IR) was recorded on a NICOLET iS10 Fourier transformed infrared spectrophotometer. The thermal stability of the products (10 mg) was measured using a TG/DTA 6200 (Japan Seiko Instruments) by heating from 50–800 °C at a heating rate of 10 °C min^−1^ under N_2_ flow (50 mL min^−1^). Glass transition temperature (*T*_g_) was characterized using a NETZSCH DSC 200 PC analyser (differential scanning calorimeter measurements) under N_2_ atmosphere. The temperature was over a range of −50 to +120 °C with a heating of 10 °C min^−1^. Approximately 10 mg of PVC materials were weighed and sealed in a 40 μL aluminum crucible and immediately detected using DSC measurement. The DSC data was collected from a first cycle of heating.^25,26^

## Conclusions

In this paper, thiophenol or long carbon chain groups in the presence of an inorganic base (K_2_CO_3_) and an organic base (DIEA) were successfully employed to remove the Cl in PVC, which convert waste PVC into value-added products to improve its plasticity for feedstock recycling. The maximum substitution ratio reached up to 35.7% when using thiophenol in the presence of DIEA, which was probably attributed to the easily formed nucleophilic group (C_6_H_5_S–). Meanwhile, the elimination ratio was just 4.4% due to the use of a weak base. It was observed that there was a slight change in the thermal stability of all the modified PVC during the first degradation stage when compared to pure PVC, which suggested that the main chemical property of PVC remained after chemical modification. Moreover, it is feasible to improve plasticity of PVC *via* replacing some Cl atoms by DINP moieties reagents because the *T*_g_ of all the modified PVCs in this work were less than that of PVC.

## Conflicts of interest

There are no conflicts to declare.

## Supplementary Material

RA-009-C9RA05081G-s001
