# Supplementary material for: Degradation of PVC waste into a flexible polymer by chemical modification using DINP moieties
Source: RSC Adv. 2019 Sep 13;9(49):28870–5. doi: 10.1039/c9ra05081g (PMC9071209; doi:10.1039/c9ra05081g)
Supplement: RA-009-C9RA05081G-s001 [file RA-009-C9RA05081G-s001.pdf]

Electronic Supplementary Material (ESI) for RSC Advances.
This journal is © The Royal Society of Chemistry 2019

Supplementary information

Degradation of PVC waste into flexible polymer by chemical modification using DINP moieties

Lihui Lu[a], Shogo Kumagai[a], Tomohito Kameda[a], Ligang Luo[b], Toshiaki Yoshioka[a]**

[a] Graduate School of Environmental Studies, Tohoku University, 6-6-07 Aoba, Aramaki-aza, Aoba-ku, Sendai, Miyagi 980-8579, Japan

[b] College of Life Science, Shanghai Normal University, 100 Guilin Road, Shanghai 200234, China

*[a] Corresponding Authors: yoshioka@env.che.tohoku.ac.jp, Tel: +81-22-795-7212; Fax: +81-22-795-7212; *[b] Corresponding author:luo_ligang@yahoo.com

12 pages

11 figures

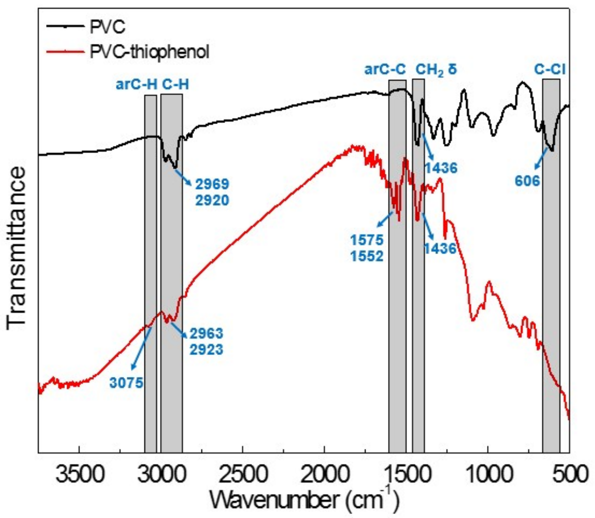

Fig. S1. FT-IR spectra of PVC and PVC-thiophenol. (Reaction condition: 500 mg PVC, 1.0 eq. thiophenol, 0.1 eq. K_2CO_3, 40°C, 50 mL DMF as solvent, 3 h.)

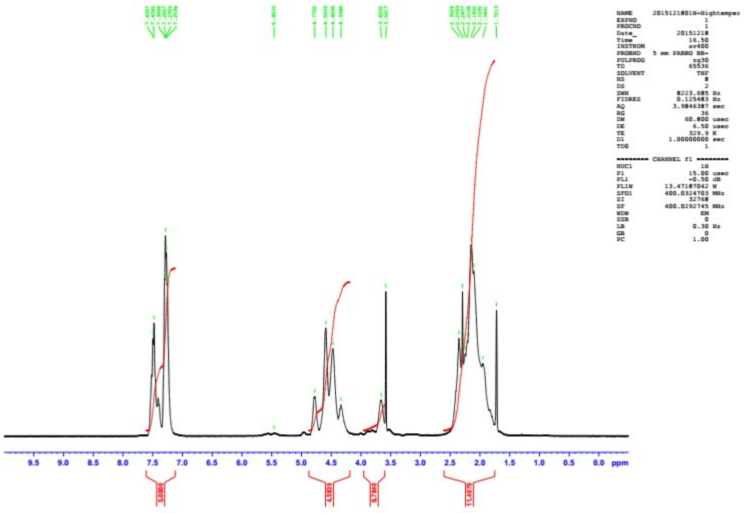

Fig. S2. ^{1}H NMR spectrum of the nucleophilic substitution with thiophenol in the presence of K_2CO_3 (400MHz, THF-d_8, 329.9K).

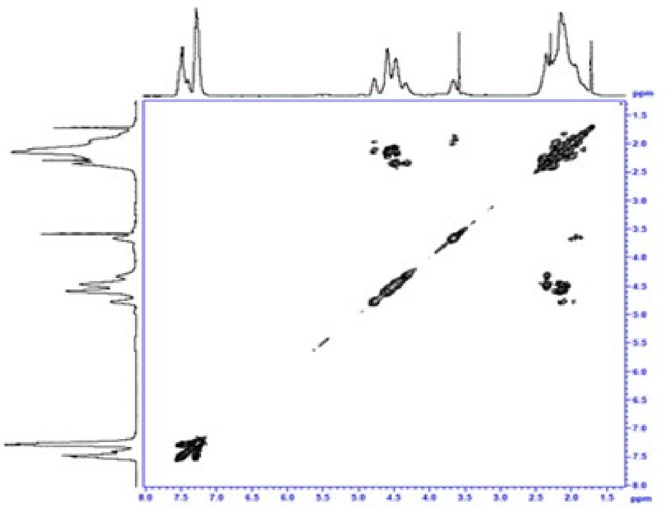

Fig. S3. ^1H-^1H COSY spectrum of the nucleophilic substitution with thiophenol in the presence of K_2CO_3 (400MHz, THF-d_8, 329.2K).

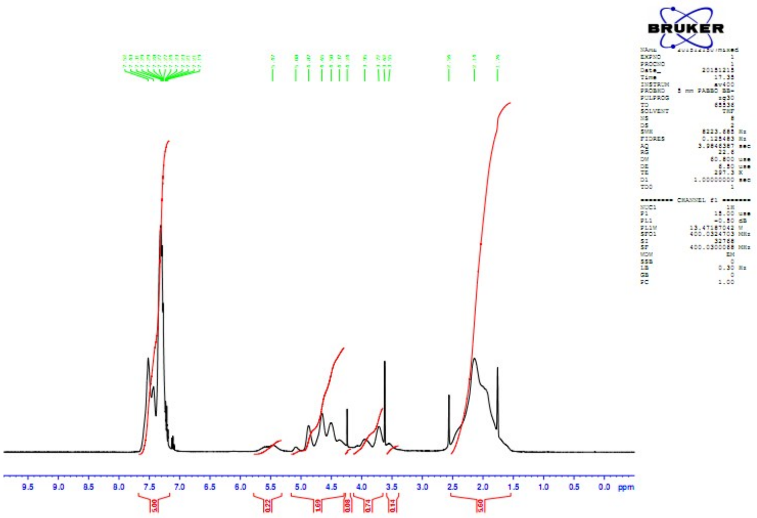

Fig. S4. ^{1}H NMR spectrum of the nucleophilic substitution with thiophenol in the presence of DIEA (400MHz, THF-d_8, 297.3K).

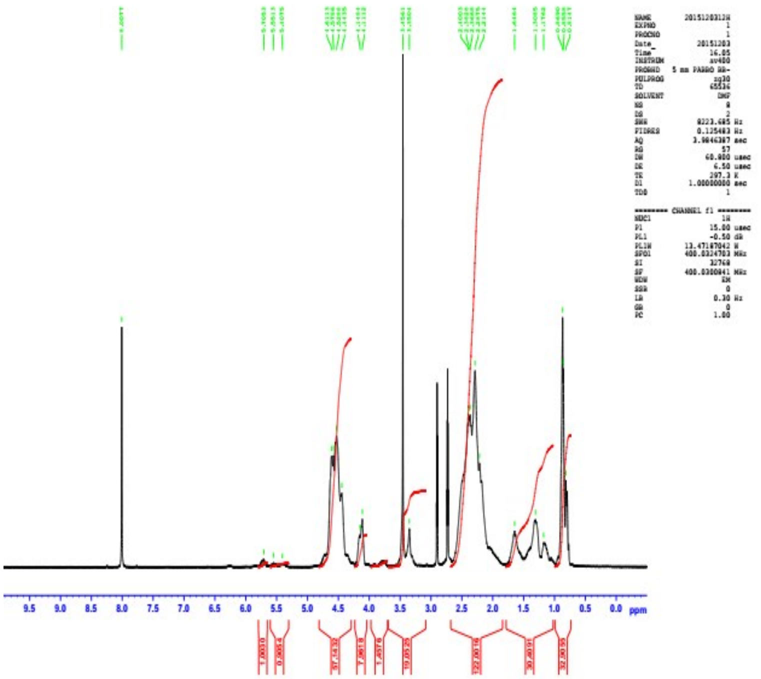

Fig. S5. ^{1}H NMR spectrum of the nucleophilic substitution with isooctyl thioglycolate in the presence of K_2CO_3 (400MHz, DMF-d$_7$, 297.3K).

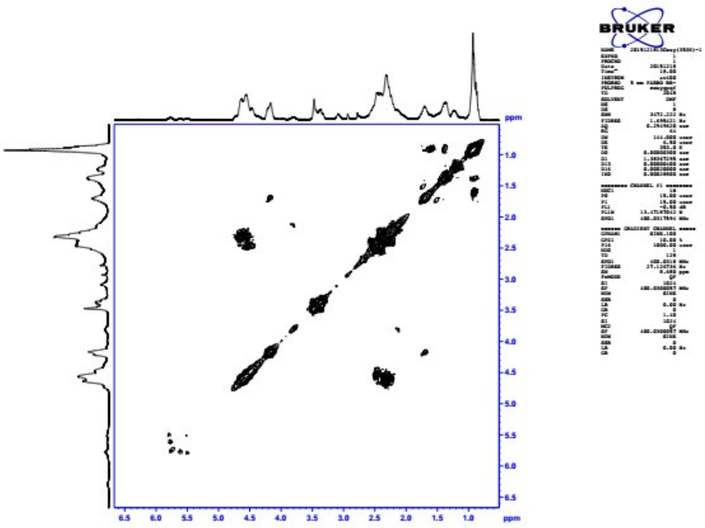

Fig. S6. ^1H-^1H COSY spectrum of the nucleophilic substitution with isooctyl thioglycolate in the presence of K_2CO_3 (400MHz, DMF-d_7, 353.0K).

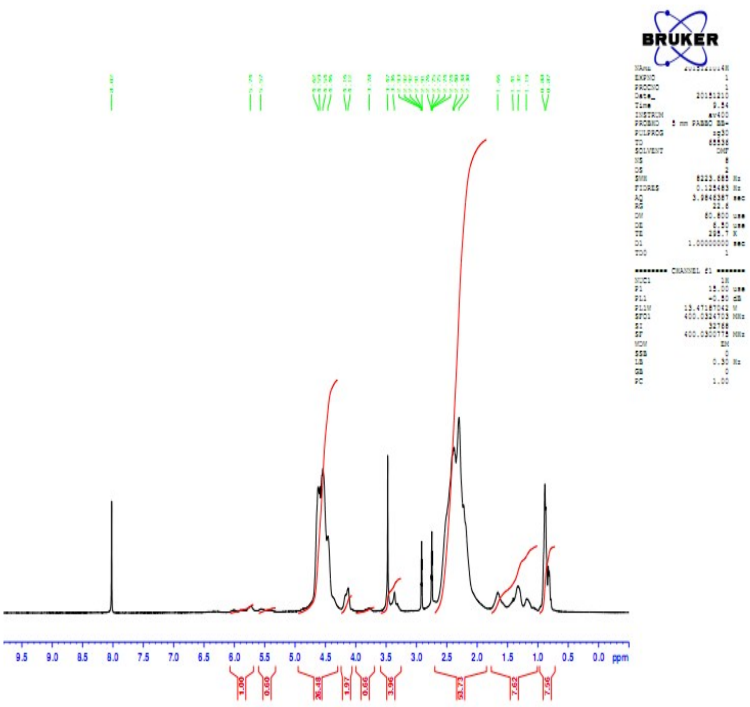

Fig. S7. ^{1}H NMR spectrum of the nucleophilic substitution with isooctyl thioglycolate in the presence of DIEA (400MHz, DMF-d_7, 295.7K).

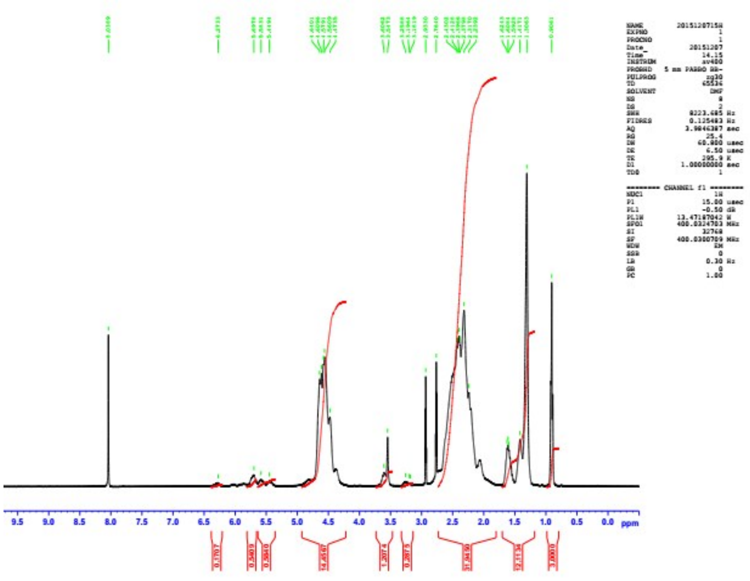

Fig. S8. ¹H NMR spectrum of the nucleophilic substitution with 1-octanethiol in the presence of K_2CO_3 (400MHz, DMF-d_7, 295.9K).

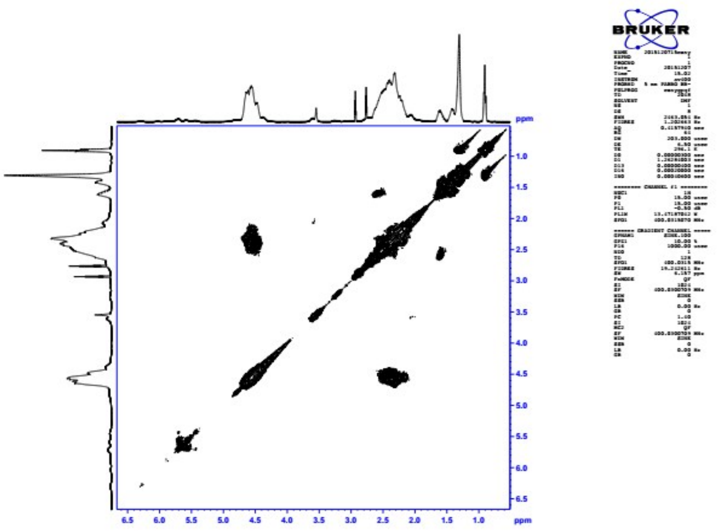

Fig. S9. ^1H-^1H COSY spectrum of the nucleophilic substitution with 1-octanethiol in the presence of K_2CO_3 (400MHz, DMF-d_7, 296.1K).

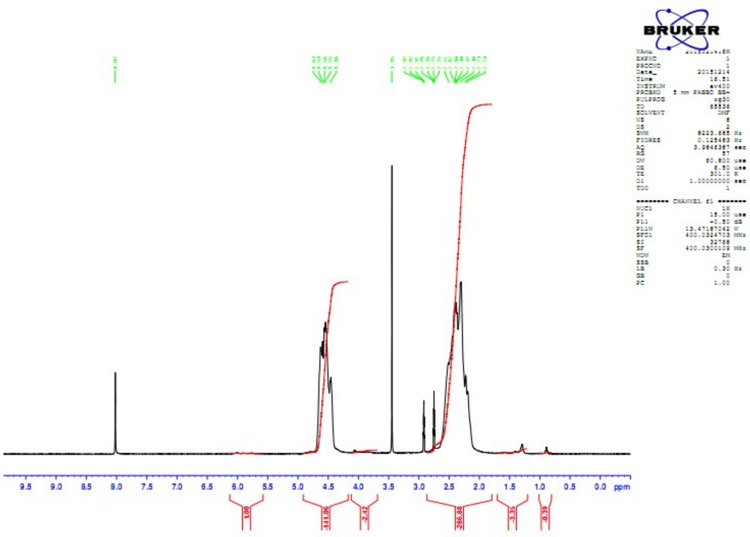

Fig. S10. ^{1}H NMR spectrum of the nucleophilic substitution with 1-Octanethiol in the presence of DIEA (400MHz, DMF-d_7, 301.0K).

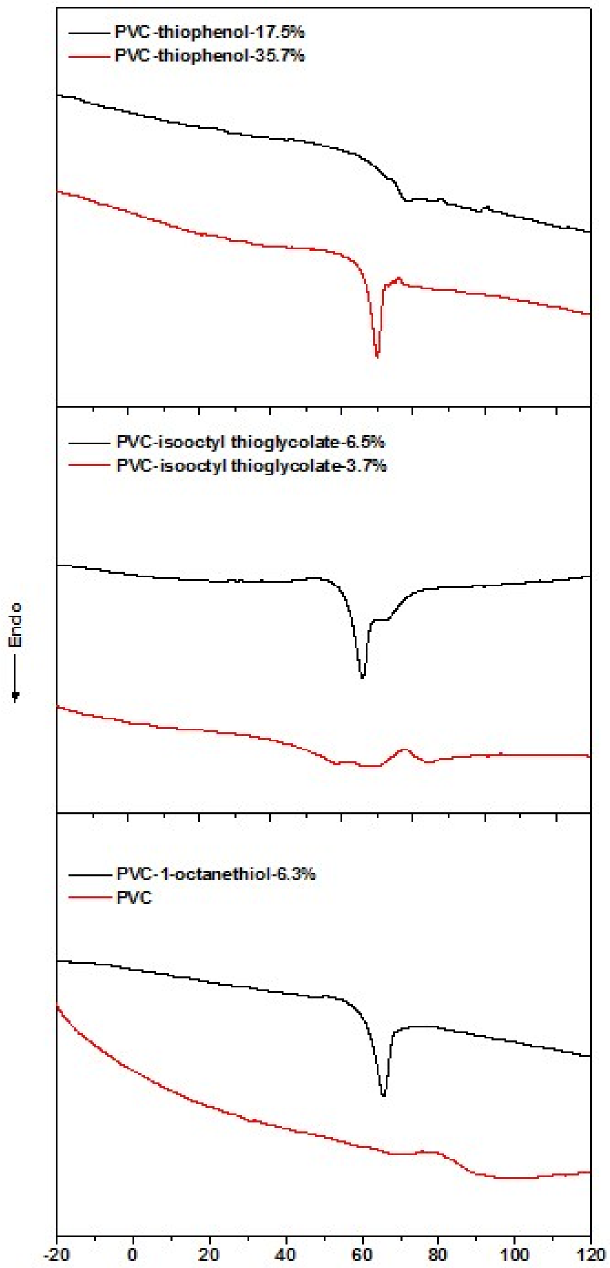

Fig. S11. DSC curves of PVC and modified PVC materials.
